# Neurite Mistargeting and Inverse Order of Intraretinal Vascular Plexus Formation Precede Subretinal Vascularization in *Vldlr* Mutant Mice

**DOI:** 10.1371/journal.pone.0132013

**Published:** 2015-07-15

**Authors:** Verity Johnson, Mengqing Xiang, Zhe Chen, Harald J. Junge

**Affiliations:** 1 Department of Molecular, Cellular, and Developmental Biology, University of Colorado, Boulder, Colorado, 80309, United States of America; 2 Center for Advanced Biotechnology and Medicine and Department of Pediatrics, Rutgers University-Robert Wood Johnson Medical School, Piscataway, New Jersey, 08901, United States of America; 3 State Key Laboratory of Ophthalmology, Zhongshan Ophthalmic Center, Sun Yat-sen University, 54 South Xianlie Road, Guangzhou, 510060, China; Children's Hospital Boston, UNITED STATES

## Abstract

In the retina blood vessels are required to support a high metabolic rate, however, uncontrolled vascular growth can lead to impaired vision and blindness. Subretinal vascularization (SRV), one type of pathological vessel growth, occurs in retinal angiomatous proliferation and proliferative macular telangiectasia. In these diseases SRV originates from blood vessels within the retina. We use mice with a targeted disruption in the *Vldl-receptor (Vldlr*) gene as a model to study SRV with retinal origin. We find that *Vldlr* mRNA is strongly expressed in the neuroretina, and we observe both vascular and neuronal phenotypes in *Vldlr^-/-^* mice. Unexpectedly, horizontal cell (HC) neurites are mistargeted prior to SRV in this model, and the majority of vascular lesions are associated with mistargeted neurites. In *Foxn4^-/-^* mice, which lack HCs and display reduced amacrine cell (AC) numbers, we find severe defects in intraretinal capillary development. However, SRV is not suppressed in *Foxn4^-/-^;Vldlr^-/-^* mice, which reveals that mistargeted HC neurites are not required for vascular lesion formation. In the absence of VLDLR, the intraretinal capillary plexuses form in an inverse order compared to normal development, and subsequent to this early defect, vascular proliferation is increased. We conclude that SRV in the *Vldlr^-/-^* model is associated with mistargeted neurites and that SRV is preceded by altered retinal vascular development.

## Introduction

Retinal vascular diseases are a leading cause of impaired vision and blindness. The subretinal space contains photoreceptor segments, and is the target of harmful neovascularization in several vision threatening pathologies, including retinal angiomatous proliferation (RAP). RAP is characterized by subretinal vascularization (SRV) that originates from intraretinal capillaries [1], and is estimated to occur in 8–22% of individuals initially diagnosed with exudative AMD [2].

Very low-density lipoprotein receptor knockout (*Vldlr* ^*-/-*^) mice provide a model system to study SRV with retinal origin. Mice exhibit a retinal vascular organization that is similar to humans. A superficial vascular plexus resides on the inner surface of the retina and two additional intraretinal vascular plexuses are embedded in the inner plexiform layer (IPL) and outer plexiform layer (OPL) [3]. *Vldlr* ^*-/-*^ mice develop vascular lesions that extend from capillaries in the OPL into the subretinal space [4]. At late stages, *Vldlr* ^*-/-*^ vascular lesions are associated with Müller glia activation, retinal rosette formation, inflammation, vascular leakage, altered growth factor expression [5–10], and reactive oxygen species accumulation [11–14]. These changes (with the exception of reactive oxygen species accumulation) occur focally in areas directly adjacent to vascular lesions.

VLDLR is a multi-functional single-pass transmembrane protein. It is a member of the low-density lipoprotein receptor (LDLR) family of endocytic receptors, functions as a receptor for triglyceride-rich lipoproteins [15], and mediates neuronal positioning in the cerebral cortex and cerebellum through Reelin/Dab-1 signaling [16]. In humans, VLDLR has been associated with an increased risk of developing AMD, [17] but the role of VLDLR in SRV remains incompletely defined.

How retinal neurons and glia instruct angiogenesis is not well understood [18]. In this study we focused on horizontal cells (HCs), a type of interneuron that displays intimate contact with retinal capillaries in the OPL (a schematic representation of the retina and its cell types is provided in S1 Fig). HCs modulate photoreceptor/bipolar cell neurotransmission in visual processing [19]. During retinogenesis HC cell bodies migrate to the outer edge of the inner nuclear layer (INL), transiently extend vertical neurites towards both the apical and basal retinal surfaces, then remodel these nascent processes into a laterally-oriented network in the OPL during the first postnatal week [20]. HC neurite mistargeting into the outer nuclear layer (ONL) is observed when photoreceptor neurotransmission or Semaphorin6A/PlexinA4 signaling is impaired (see discussion).

Here, we report the unexpected finding that HC neurites are mistargeted into the ONL and subretinal space prior to SRV in *Vldlr* ^*-/-*^ mice, and that most vascular lesions are associated with mistargeted neurites. We find that *Vldlr* mRNA is highly expressed in the neuroretina, suggesting a role for retinal neurons in the *Vldlr* ^*-/-*^ SRV phenotype. We use Foxn4 knockout (*Foxn4* ^-/-^) mice, which completely lack HCs and display reduced numbers of ACs, to demonstrate the importance of interneurons in normal retinal angiogenesis. We then use *Foxn4* ^-/-^ *;Vldlr* ^*-/-*^ double knockout mice to show that SRV can occur in the absence of HCs. Our analysis of early postnatal development establishes that SRV in *Vldlr* ^*-/-*^ mice is preceded by developmental defects including an inverse order of intraretinal capillary plexus formation and a subsequent increase in vascular cell proliferation.

## Materials and Methods

### Animals

Mice heterozygous for the *Vldlr* ^*tm1Her*^ mutation were obtained from the Jackson laboratory (strain B6;129S7-*Vldlr* ^*tm1Her*^/J) and maintained on a C57BL/6J background. *Vldlr* mice were genotyped using a set of three primers: a forward primer upstream of the targeting cassette (5'-TGGTGATGAGAGGCTTGTATGTTGTC-3'), a reverse primer within the targeting cassette (5'-CCAGCTGGGGCTCGATCGAG-3'), and a reverse primer downstream of the targeting cassette (5'-TTGACCTCATCGCTGGCGGCCTTG-3'), this set amplifies a 148 bp product for the mutant allele and a 461 bp product for the WT allele. Mice heterozygous for *Foxn4* were generated previously [21], maintained on a C57BL/6J background, and genotyped as described [22]. In order to improve postnatal viability of both *Foxn4* ^*-/-*^ and *Foxn4* ^*-/-*^ *;Vldlr* ^*-/-*^ pups, these mice were bred on a mixed CD1 and C57BL/6J background. Mice were euthanized using a CO_2_ chamber and subsequent cervical dislocation, or using an isoflurane drop jar and subsequent decapitation. We observed that postnatal retinal angiogenesis progressed slightly faster than expected in all mouse lines kept at the animal facility in Boulder. We attributed this difference to a lower oxygen concentration at 5500 ft elevation. All experiments used mice of either sex.

### Fluorescent immunostaining

Tissues were fixed for 3 hours in 10% neutral buffered formalin (NBF) on ice. For whole mount preparations, retinas were dissected in PBS. For retinal sections, eyeballs were immersed in PBS and opened by incising the cornea. Eyeballs were then cryoprotected in 30% sucrose, PBS at 4°C overnight, embedded in a cryomold filled with O.C.T. compound (Tissue-Tek), and rapidly frozen on dry ice. Frozen specimens or sections were stored at -80°C until use. For whole mount immunostaining, retinas were blocked and permeabilized in PBS, 0.5% TritonX-100 (0.5% PBST) with 5% goat serum for 2–4 hours at RT, then incubated overnight at 4°C with primary antibody diluted in blocking buffer. Retinas were then washed 5 times for 1 hour at 25°C with 0.5% PBST, and incubated overnight with the appropriate secondary antibody and DAPI (1 μg/ml) in blocking buffer at 4°C (Alexa Fluor 488 or Alexa Fluor 555 anti-IgG (H+L) antibodies, Invitrogen, dilution 1:800). The retinas were then washed again as above, post-fixed for 5 min in 10% NBF, washed 3x with PBS and mounted in Fluoromount-G (Southern Biotech). For immunostaining of retinal sections, slides were warmed to 37°C, blocked and permeabilized in 0.1% PBST with 5% goat serum for 40 min at RT, then incubated with primary antibodies diluted in blocking buffer overnight at 4°C. Slides were then washed with 0.1% PBST 3 times for 2 min, incubated with the appropriate secondary antibody for 1 hour at RT, washed 3 times for 2 min in 0.1% PBST, post-fixed, and mounted as described above. The following primary antibodies were used for this study: CD31 1:50 (BD Biosciences, 550274), Isolectin GS-IB4 Alexa Fluor 488 conjugate 1:100 (Invitrogen, I21411), Pals1 1:800 (Millipore, 07–708), GFAP 1:400 (DAKO, Z033429), F4/80 1:350 (AbD Serotec, MCA497R), PLVAP 1:200 (BD Biosciences, 550563), Neurofilament-L 1:100 (Cell Signaling, 2837), Calbindin 1:2500 (Millipore, AB1778), PSD95 1:500 (Cell Signaling, 3450), Calretinin 1:2000 (Millipore AB1550), ChAT 1:200 (Millipore AB144P), Syt2b 1:7500 (ZIRC, zpn-1), VGLUT3 1:2500 (Millipore, AB5421), Tyrosine Hydroxylse 1:1000 (Millipore, AB152), Melanopsin 1:2500 (Advanced Targeting Systems, AB-N38), Neuropeptide Y 1:3200 (Millipore, 11976P), Sox9 1:2000 (Millipore, AB5535), PKCalpha 1:250 (Santa Cruz, sc-208), Recoverin 1:1000 (Millipore, AB5585). Extravasated mouse IgG was detected using Alexa 555 coupled goat anti-mouse antibody (Invitrogen, A21424).

### Expression of mVLDLR constructs in HeLa cells

Image clone 3968213 was obtained and PCR was used to subclone the coding sequence with a c-terminal HA-tag into XbaI and HindIII site of an existing modified pcDNA3.3 vector. To generate pcDNA3.3 mVLDLRΔexon5-HA, total RNA from a *Vldlr* ^-/-^ retina was reverse transcribed into cDNA (Roche High Fidelity RT). An N-terminal fragment of the mutant message in which exon5 is skipped was subcloned into pcDNA3.3 mVLDLR-HA using XbaI and ApaI sites. Constructs were verified by sequencing. HeLa CCL-2 cells cultured in 4-well chamber slides (LabTek) were transfected with 400 ng mVLDLR-HA or mVLDLRΔexon5-HA constructs using Lipofectamine 2000 (Invitrogen). After 48 hours of expression, wells were fixed in 10% NBF for 5 minutes at 25°C and processed for immunostaining as described above, or used for branched DNA *in situ* hybridization assays.

### Branched DNA in situ hybridization

Retinal tissue sections were prepared as described above. 12 μm sections at the level of the optic nerve head were collected, stored at -20°C, and assayed for *Vldlr* expression the following day. The assay was performed according to manufacturer instructions (QuantiGene ViewRNA ISH Tissue 1-Plex Assay Kit, Affymetrix). Branched DNA probes were from Affymetrix (m*vldlr*_ex5: VB1-16261) and were used at a 1:50 dilution. Immunostaining was performed subsequent to in situ hybridization.

### Quantification of mistargeted neurites and vascular lesions

Sixteen (P9) or twenty-four (P14, P21) uninterrupted 20 μm serial sections surrounding the optic nerve head were quantified per retina for 3 *Vldlr* ^*-/-*^ and 3 WT retinas. In order to avoid counting a single vascular lesion multiple times, lesion maps were created by imaging each isloectinB4 stained retinal section and then tracking each vascular lesion throughout the series of sections. A vascular lesion was counted as neurite-associated (NF^+^) if it was in contact with one or more mistargeted neurites. An HC neurite was counted as mistargeted only if it extended more than halfway across the width of the ONL.

### Administration and staining of EdU

An EdU stock solution was prepared by dissolving 1 mg of EdU (Invitrogen, A10044) in 10 μl DMSO and 90 μl PBS. Animals were intraperitoneally injected with 10 μl EdU stock solution per gram body weight 3 hours prior to sacrifice. Whole eyes were then harvested and fixed in 10% NBF for 3 hours on ice. Immunostaining of retinal whole mounts was performed as described above using Alexa Fluor 488 conjugated anti-CD31 or isolectinB4. After staining with a vascular marker, retinas were washed 3 times for 1 hour with 0.5% PBST and EdU positive cells were identified using the Click-iT EdU Alexa Fluor-555 Imaging kit (Invitrogen, C10338) according to manufacturers instructions.

### Quantification of EdU^+^ vascular cells and quantification of vertical vessels in the INL

For quantification of EdU positive cells in the superficial vascular plexus at P6, 4 petals were imaged per retina using confocal microscopy. The number of EdU/IsolectinB4 double positive vascular cells was then counted in one 500 μm^2^ area per petal (four petals were averaged per retina). 3 whole mount specimens were analyzed per group.

For quantification of EdU positive cells in the superficial, intermediate, and deep vascular plexuses at P14, 4 petals were imaged per retina close to the optic nerve head (one 636 μm^2^ area per petal, 35 optical sections of 0.91 μm per stack). Separate Z-projections were generated for the superficial, intermediate and deep vascular plexuses and EdU/IsolectinB4 double positive vascular cells were counted in each vascular layer. The NFL and IPL were quantified side-by-side to ensure each cell was only counted once. The number of proliferating vascular cells was averaged per retina and 4 retinas were analyzed per group. To quantify the number of lesions with EdU positive vascular cells at the base, one 636 μm2 area near the optic nerve head was imaged per retina (n = 4) from the OPL through the lesion heads (40 optical sections of 0.91 μm per stack). Lesions were counted as EdU positive if an EdU positive cell was present in a 10 μm radius around the lesion base.

The number of INL-spanning vertical vessels at P14 was counted on the same whole mounts used for EdU quantification by manually scanning through the entire stack to identify vertical vessels that span the INL to join the deep vascular plexus in the OPL.

### Vascular branch-point quantification

For quantification of branch points in the deep vascular plexus at P14, 4 petals were imaged per retina and confocal z-projections of the OPL were generated for 600 μm^2^ areas within 200 μm of the optic nerve head (central) or 200 μm of the rim of the retina (periphery). The number of branch points was counted manually, 3–4 specimens were analyzed per group.

### Imaging

Images of retinal sections were taken on an epifluorescent Leica DM IL LED Microscope using QCapture Pro 6.0 imaging software. Images of retinal whole mounts (except where indicated) were taken on a Zeiss 510 LSM confocal microscope using ZEN imaging software. All images were processed using Fiji software, and figures were prepared using Adobe Photoshop and Adobe Illustrator software.

### Statistics

Graphs are shown as mean ± standard deviation. Statistical analyses were performed using two-tailed, heteroscedastic Student’s *t*-tests and p-values less than 0.05 were considered statistically significant.

### Study approval

All animal use for this study was approved by the Institutional Animal Care and Use Committee (IACUC) at the University of Colorado Boulder (protocol 1307.04). Animal experimentation was conducted in accordance with federal animal experimentation guidelines and all efforts were made to minimize animal suffering.

## Results

### Outer limiting membrane disruption is a key step in the progression from early to late stage phenotypes in *Vldlr* ^*-/-*^ retinas

**Fig 1:**
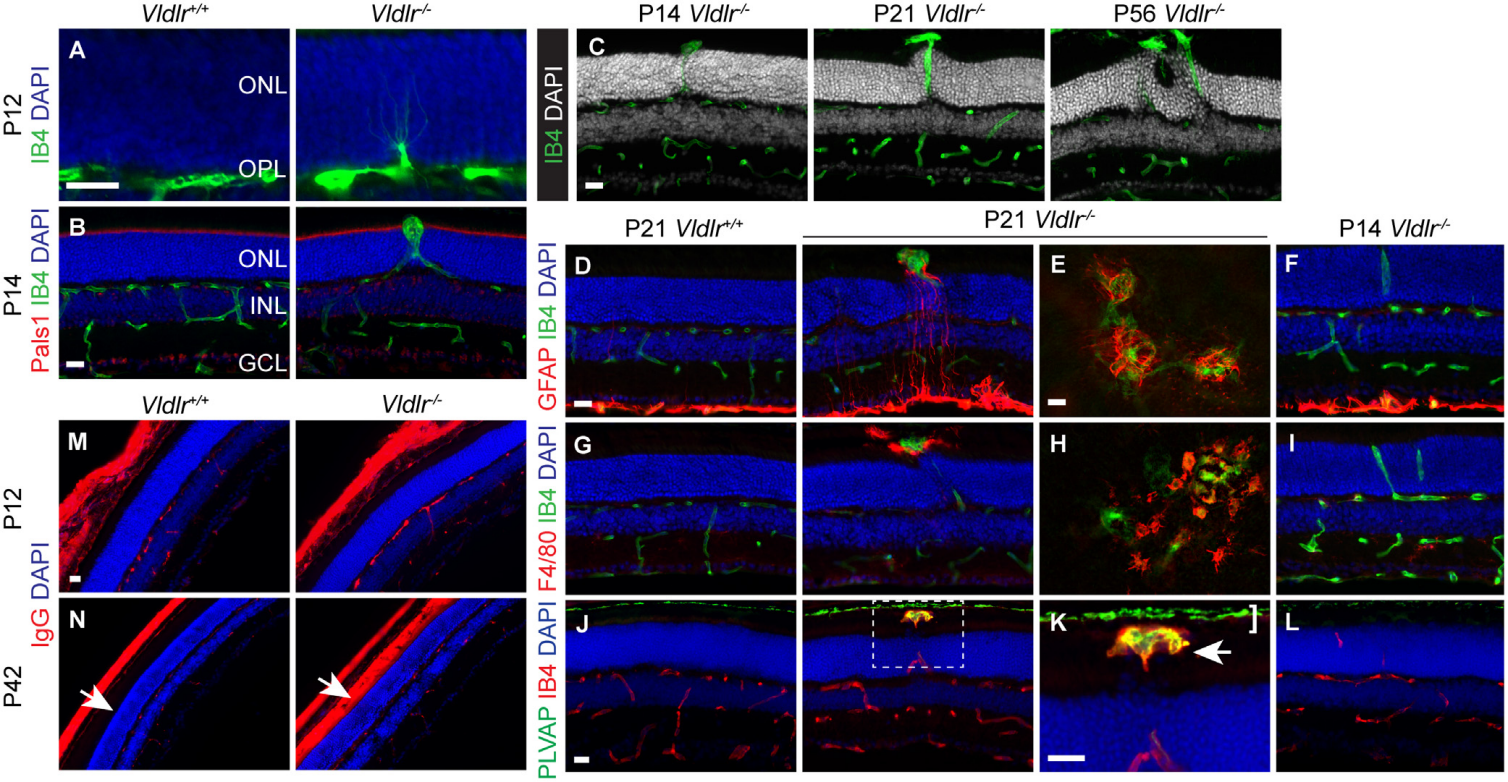
Secondary phenotypes triggered by vascular lesions and outer limiting membrane disruption. (**A**) Early vascular lesion formation in isolectinB4 stained P12 retinal sections. Endothelial cells invade the ONL from the OPL capillary bed. (**B**) Vascular lesions penetrate the Pals1 positive OLM by P14. (**C**) Retinal rosettes develop subsequent to OLM disruption. (**D-F**) Müller glia activation marked by GFAP staining in P21 retinal sections and whole mounts (**E**) imaged from the outer retinal side. GFAP is focally upregulated adjacent to vascular lesions. GFAP expression is normal at P14, the dense GFAP staining in the inner retina is from GFAP positive astrocytes. (**G**) At P21 F4/80 positive macrophages cluster in *Vldlr* ^-/-^ lesion heads, (**H**) similar macrophage accumulation is also seen on whole mounts imaged from the outer retinal side. (**I**) Macrophage distribution in P14 *Vldlr* ^-/-^ retinas appears normal. (**J**) In wild type P21 retinas, PLVAP positive endothelial cell fenestrations are present only in choriocapillaries. In the mutant retina, PLVAP is locally upregulated in vascular lesion heads. Higher magnification (**K**) shows PLVAP expression in lesion heads (arrow) and choriocapillaries (bracket). (**L**) PLVAP is not yet upregulated in P14 vascular lesions. (**M, N**) IgG extravasation is confined between the OLM and retinal pigment epithelium in the peripheral retina. Aside from lesions, the retinal vasculature does not leak IgG at P42 and no IgG extravasation is detected in P12 mutant sections. GCL, ganglion cell layer; OLM, outer limiting membrane; ONL, outer nuclear layer; OPL, outer plexiform layer. Scale bars: 20 μm.

Previous characterizations of *Vldlr* ^*tm1Her/tm1Her*^ (*Vldlr* ^*-/-*^) mice have revealed the involvement of several retinal cell types at vascular lesion sites including photoreceptors, Müller glia, and macrophages [5, 8, 11]. However, the temporal sequence of phenotypic changes remained unclear. To determine which retinal cell types exhibit phenotypes first, we systematically analyzed early postnatal time points. We found that the onset of vascular lesion formation was at postnatal day 12 (P12) when multiple isolectinB4 positive endothelial cells invaded the normally avascular ONL predominantly in the central retina (Fig 1A). By P14, numerous angiogenic sprouts penetrated the outer limiting membrane (OLM), a belt of adherens junctions between photoreceptors and Müller glia (Fig 1B). Staining for the OLM subapical region component PALS1 (Protein associated with Lin7) revealed that vascular lesions locally disrupted the OLM, while it appeared intact in adjacent areas (Fig 1B) and prior to penetration by SRV. Disruption of the OLM could trigger key secondary changes because it provides mechanical strength to the retina and is involved in maintaining photoreceptor cell polarity [23]. Loss of proteins required for photoreceptor cell polarity and OLM adherens junction integrity, (e.g., *Crumbs2* and *Pals1*), leads to the formation of retinal rosettes and reactive gliosis [24, 25]. Indeed, we observed the formation of retinal rosettes and reactive gliosis in *Vldlr* ^*-/-*^ retinas, but only after the OLM was disrupted by SRV. While retinal rosettes were usually absent in immature P14 lesions, progressive rosette formation was observed at P21 and P56 (Fig 1C). In addition, we detected focal upregulation of the reactive gliosis marker glial fibrillary acidic protein (GFAP) in *Vldlr* ^*-/-*^ retinas at P21 but not at P14. Analysis of GFAP-stained P21 whole mounts showed that Müller glia had infiltrated vascular lesion heads and thus exhibited an abnormal localization in the subretinal space (Fig 1D–1F). We also detected the accumulation of F4/80 positive macrophages in vascular lesion heads at P21 but not at P14 (Fig 1G–1I). Rosettes, reactive gliosis, Müller glia mislocalization, and macrophage recruitment only occurred in locations where aberrant neovessels had penetrated the OLM, thus they are likely consequences of local OLM disruption.

To maintain the integrity of the inner blood-retina barrier, retinal endothelial cells are not fenestrated and express very low levels of plasmalemma vesicle–associated protein (PLVAP), a component of endothelial cell fenestrations. Interestingly, we found that PLVAP was upregulated in vascular lesion heads in the subretinal space at P21, while it was not significantly expressed in any other part of the retinal vasculature in *Vldlr* ^*-/-*^ mice. PLVAP was not upregulated in *Vldlr* ^*-/-*^ retinas at P14, suggesting that defective blood-retina barrier formation is not involved in early stages of vascular lesion formation (Fig 1J–1L). Consistent with the presence of aberrant endothelial cell fenestrations in vascular lesion heads we found massive extravasation of mouse IgG into the subretinal space and interphotoreceptor matrix in *Vldlr* ^*-/-*^ retinas at P42 but not at P12 (Fig 1M and 1N). Importantly, we found that blood vessels in other retinal layers (in the NFL, IPL, and OPL) did not leak substantial amounts of IgG.

Our analysis of the temporal sequence of vascular lesion formation in *Vldlr* ^*-/-*^ mice extends earlier phenotypic characterizations and shows that OLM disruption is a key step in the progression from early SRV to Müller glia activation, macrophage recruitment, and vascular leakage.

### VLDLR is broadly expressed in the neuroretina

To define which retinal cell types express *Vldlr*, we localized *Vldlr* mRNA using a branched DNA (bDNA) signal amplification in situ hybridization assay [26]. We employed a probe set that consists of 6 pairs of bDNA oligonucleotides targeted to the portion of exon5 that is deleted in the *Vldlr* ^*tm1Her*^ allele (probe m*vldlr*_ex5) [27]. To validate this probe we expressed full-length and exon5-deficient VLDLR (mVLDLR-HA and mVLDLRΔexon5-HA) in Hela cells. Staining with anti-HA antibody confirmed that mVLDLR-HA and mVLDLRΔexon5-HA mRNA and protein were present, however as expected, we observed a strong in situ hybridization signal only in cells expressing mVLDLR-HA (S2 Fig).

**Fig 2:**
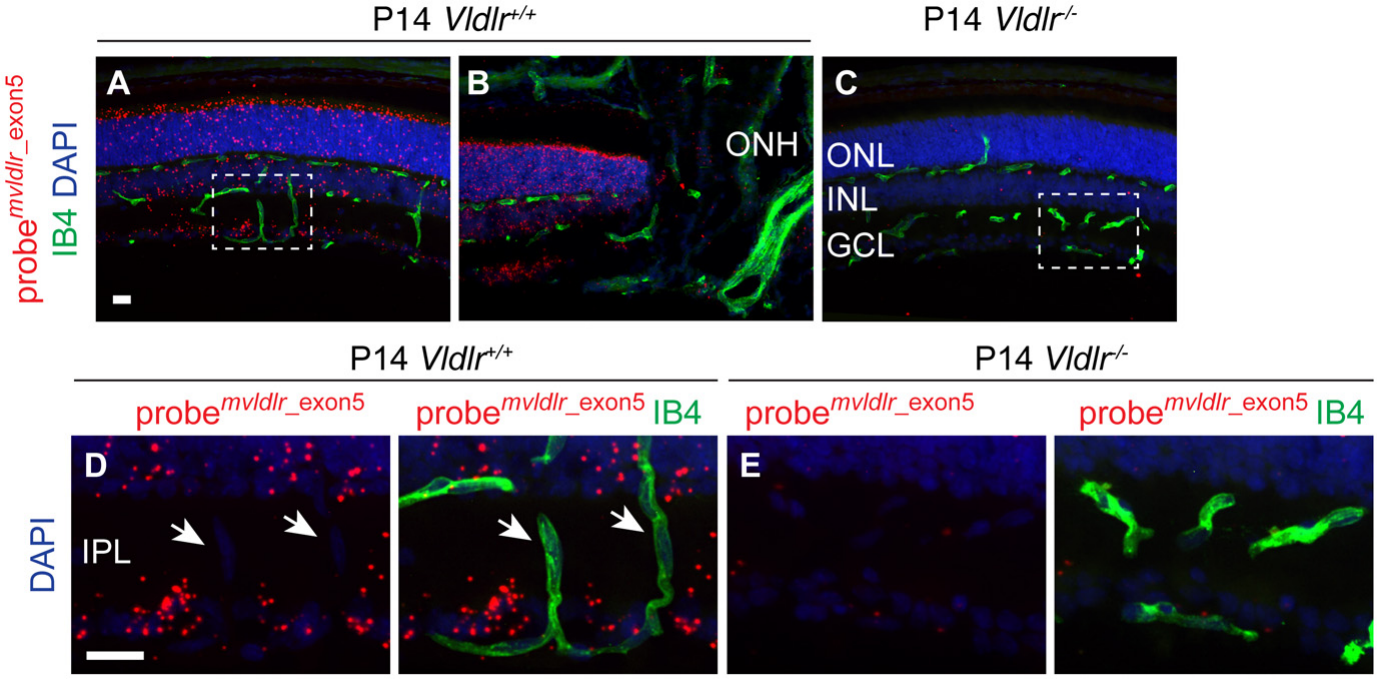
VLDLR is broadly expressed in the neural retina. (**A**) *In situ* hybridization using a bDNA probe set that hybridizes to a portion of exon5 that is not present in *Vldlr* ^-/-^ mice due to homologous recombination. The probe set reveals broad expression in the three nuclear layers of the retina. (**B**) No substantial *Vldlr* expression is detected in isolectinB4 stained retinal endothelial cells or in the central retinal artery extending from the optic nerve head. (**C**) *Vldlr* ^-/-^ tissue was used as a specificity control. (**D, E**) Enlargement of the boxed areas shows that IPL-spanning capillaries (arrows) display no substantial *Vldlr* expression. ONH, optic nerve head; GCL, ganglion cell layer; ONL, outer nuclear layer; OPL, outer plexiform layer; IPL, inner plexiform layer. Scale bars: 20 μm.

In situ hybridization on wild type retinal sections at P14 revealed *Vldlr* expression in all three nuclear layers, i.e., in the ganglion cell layer (GCL), INL and ONL (Fig 2A), while the optic nerve, choroid, ciliary body, iris, and cornea reacted largely negative (Fig 2B and S2 Fig). Importantly, virtually no signal was detected in *Vldlr* ^*-/-*^ tissue (Fig 2C and 2E). The IPL contains no neuronal or glia nuclei, therefore vascular cells in IPL-spanning vessels can be analyzed in the absence of overlapping signal from other cell types. Analysis of IPL vasculature yielded no evidence for significant expression of *Vldlr* in endothelial cells (Fig 2D).

*Vldlr* expression was similarly restricted to the GCL, INL, and ONL at earlier (P8) and later (P28) timepoints (S2 Fig). The expression profile of *Vldlr* suggests that vascular lesions could develop secondary to neural defects.

### Horizontal cell neurite mistargeting precedes vascular lesion formation and most vascular lesions form along mistargeted neurites

**Fig 3:**
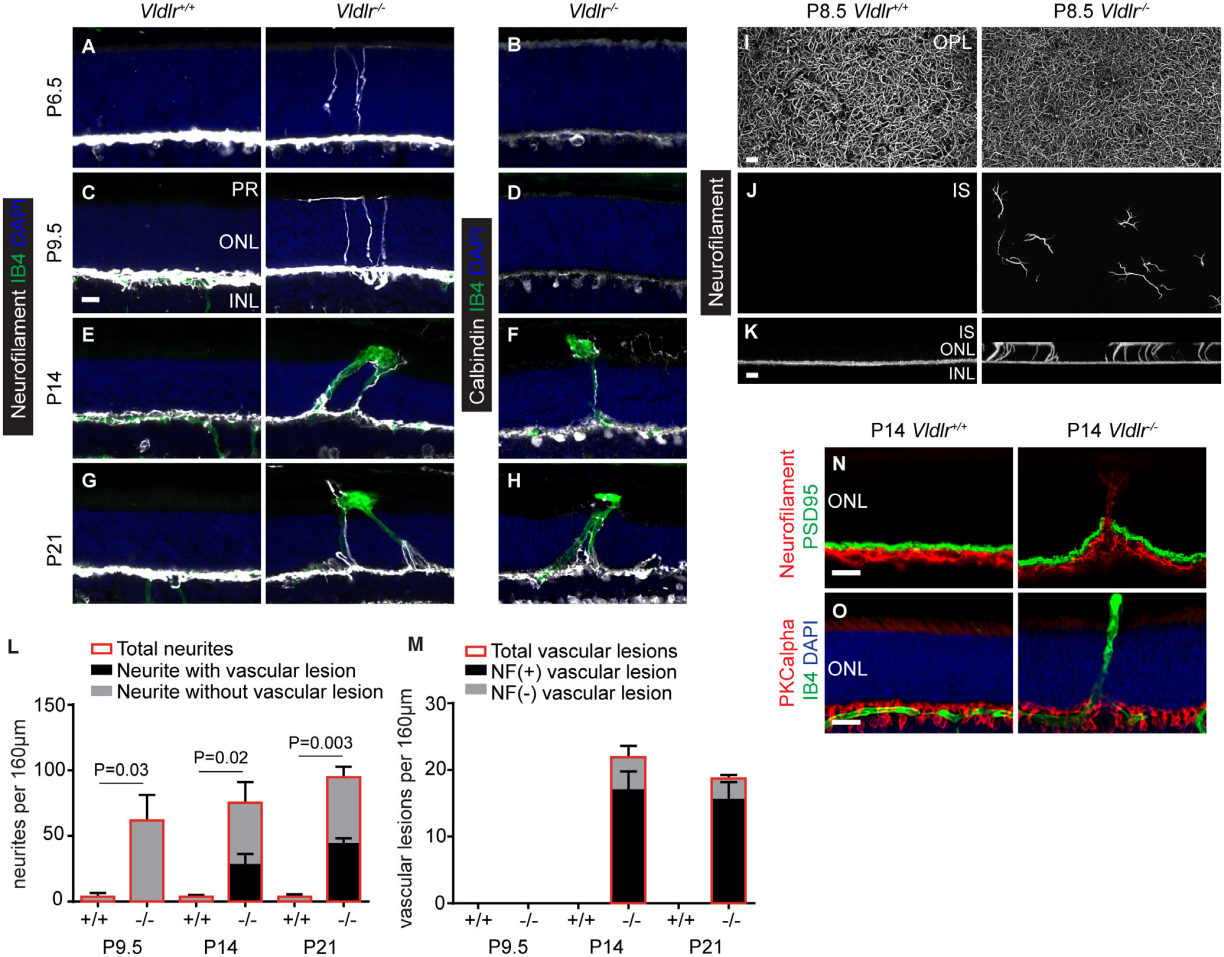
Mistargeted horizontal cell neurites are associated with vascular lesions. (**A, C**) At P6.5 and P9.5, prior to vascular lesion formation, neurofilament positive HC neurites mistarget into the ONL. (**E, G**) Vascular lesions form preferentially along mistargeted HC neurites. (**B, D, F, H**) Mistargeted HC neurites are initially not calbindin positive, but express calbindin at P14 and P21. (**I**) Confocal z-projections at the level of the OPL show the HC neurite meshwork. (**J**) Confocal z-projections at the level of photoreceptor inner segments show mistargeted, branched neurites in *Vldlr* ^-/-^ retinas. (**K**) Confocal 3D-renderings reveal that these mistarged neurites are extending from the OPL and branch out at the level of the OLM. (**L**) Mistargeted neurites and vascular lesions were quantified from 16–24 uninterrupted 20 μM serial sections per specimen (n = 3) and their number was plotted per 160 μM tissue depth. Each section was imaged and compared to neighboring sections to avoid counting vascular lesions multiple times. The total number of neurofilament positive HC neurites (bars with red outline) and the fraction of neurites that is associated with vascular lesions (black areas) as well as the fraction of neurites that is not associated with vascular lesions (grey areas) are shown. Very few mistargeted neurites were identified in WT retinas, the P-values show that the difference of total neurite number between genotypes is significant at each age (n = 3, mean +STDEV). (**M**) The total number of vascular lesions (bars with red outline), the fraction of vascular lesions associated with mistargeted neurites (black areas) and the fraction of vascular lesions not associated with mistargeted neurites (grey areas) is shown. No vascular lesions were detected in WT specimens. (**N**) Mistargeted neurites do not form PSD95 positive ectopic synapses in the ONL. (**O**) Rod bipolar cells exhibit no neurite sprouting into the ONL. INL, inner nuclear layer; IS, photoreceptor inner segments; ONL, outer nuclear layer; OPL, outer plexifom layer; PR, photoreceptor. Scale bars: 20 μm.

In order to identify potential neuronal phenotypes in *Vldlr* ^*-/-*^ retinas, we stained major retinal cell populations for appropriate markers (see Materials and Methods). Staining for neurofilament, an HC neurite marker, revealed a neurite mistargeting phenotype. At P6.5 and P9.5, WT HC neurites were laterally oriented and typically confined to the OPL, however a significant number of HC neurites in *Vldlr* ^*-/-*^ retinas aberrantly projected through the ONL (Fig 3A and 3C). The mistargeted neurites stained positive for neurofilament at all developmental stages (P6.5–P21), whereas neurites were calbindin positive only at slightly later stages (P14–P21) (Fig 3B, 3D, 3F and 3H). Confocal analysis of retinal whole mounts revealed that the dense meshwork of laterally oriented HC neurites in the OPL appeared largely unaltered in mutant retinas at P8.5 (Fig 3I). However, optical sections at the level of the photoreceptor inner segments showed mistargeted, branched neurites in the mutant retina (Fig 3J), which could be traced back to the OPL in confocal 3D-renderings (Fig 3K).

We asked if mistargeted HC neurites form ectopic synapses in the ONL, but found no postsynaptic density protein 95 (PSD-95) positive synapses associated with mistargeted neurites (Fig 3N). In addition, dendrites of another cell type participating in OPL triad synapses, rod bipolar cells, exhibited no evidence for mistargeting when stained for the rod bipolar cell marker protein kinase C alpha (PKCalpha) prior to lesion formation (Fig 3O). These findings differentiate the *Vldlr* mutant neurite mistargeting phenotype from similar phenotypes observed in mouse mutants with impaired photoreceptor glutamate release (see discussion). Because Reelin is a functional ligand for VLDLR in the context of cortical neuron migration, we asked if *Reelin* ^-/-^ mice exhibit HC neurite mistargeting. Reelin mutant mice showed occasional ectopic rod bipolar cell synapses as previously reported [28, 29], but did not display HC neurite mistargeting (S3 Fig).

Vascular lesions begin to form at P12, thus neurite mistargeting occurs well before the onset of lesion formation and may play an important role in the subsequent development of vascular phenotypes. To test the hypothesis that mistargeted neurites provide a template for vascular invasion into the ONL, we co-labeled retinal sections with isolectinB4 and neurofilament. We observed a striking overlap between mistargeted HC neurites and endothelial cells of vascular lesions (Fig 3E–3H). At P14, nascent vascular lesions were associated with one or several mistargeted neurites, and this association persisted at later time points (P21, P28, P56). Quantification of mistargeted neurites and vascular lesions from serial sections revealed that the number of mistargeted neurites increased slightly, though not significantly, from P9.5 to P21 (Fig 3L). Vascular lesions were not present at P9.5 and the number of vascular lesions remained largely constant between P14 and P21 (Fig 3M), although individual vascular lesions grew in size. The number of mistargeted neurites exceeded the number of vascular lesions in *Vldlr* ^*-/-*^ retinas, as only 47% of mistargeted neurites were lesion-associated at P21. In contrast, the vast majority of vascular lesions aligned with neurofilament-positive mistargeted neurites (83% at P21) (Fig 3L and 3M). Our finding that the majority of vascular lesions formed on mistargeted HC neurites raised the possibility that mistargeted neurites could facilitate SRV.

### Defective retinal angiogenesis in *Foxn4* ^-/-^ mice

**Fig 4:**
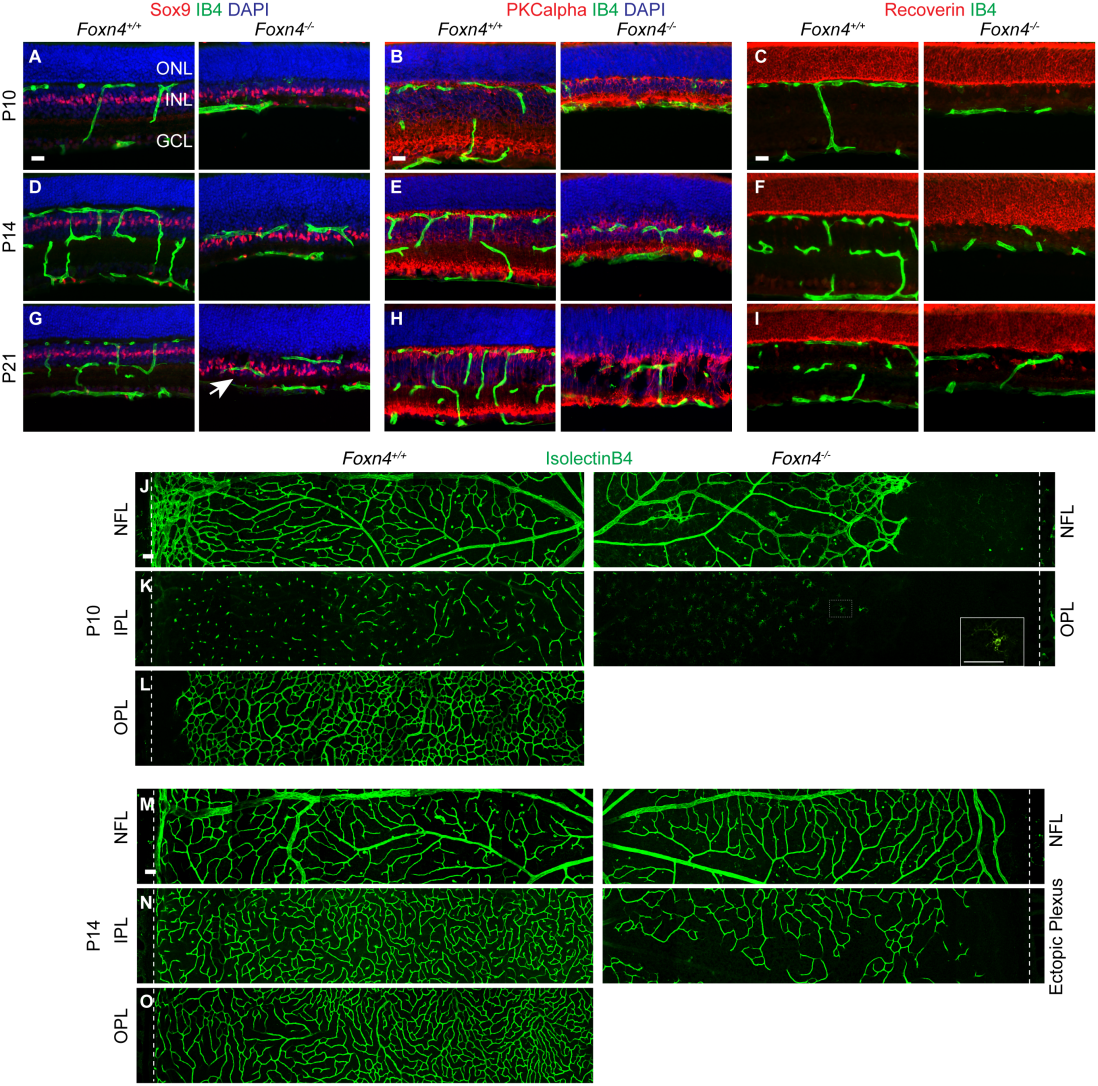
Defective intraretinal vascular development in *Foxn4* ^-/-^ mice. (**A-I**) Vascular development relative to Müller cell nuclei (Sox9), rod bipolar cells (PKCalpha), and photoreceptors (Recoverin) at the indicated time points. *Foxn4* ^-/-^ retinas display no intraretinal capillaries at P10, only sporadic isolectinB4 positive macrophages are seen within the retina. By P14, the NFL, IPL, and OPL are vascularized in WT mice, while *Foxn4* ^-/-^ mice exhibit only a single, aberrant intraretinal vascular plexus. Capillaries in this plexus are found in a range of locations, i.e., overlapping with Sox9 positive nuclei (white arrow in G), below rod bipolar cells, and in varying distances from the recoverin positive photoreceptors. Between P10 and P14 the *Foxn4* ^-/-^ retina looses lamination in the OPL region, and by P21 frequent splits in the INL portion of the retina are observed (H, right panel). Rod bipolar cell neurites sprout into the ONL. (**J-O**) Confocal z-projections of the indicated vascular layers. Panels were generated by stitching 5 projections per panel. Vascular development in the NFL is delayed at P10 in *Foxn4* ^-/-^ mice but less affected by P14. No intraretinal capillaries are present in P10 *Foxn4* ^-/-^ mice, but many isolectinB4 positive macrophages are present (inset in K shows enlarged macrophage). The ectopic intraretinal plexus is poorly developed by P14. GCL, ganglion cell layer; ONL, outer nuclear layer; IPL, inner plexiform layer; OPL, outer plexiform layer; NFL, nerve fiber layer. Scale bars: 20 μm (sections), 50 μm (whole mounts).

Despite the spatial overlap between blood vessels and HC neurites in the OPL of WT retinas, it remains unknown if HCs play important roles in retinal angiogenesis. To evaluate the role of HCs in developmental angiogenesis we turned to mouse models that lack HCs. Driving diphteria toxin receptor expression under the connexin57 promoter causes the genetic ablation of HCs in adult tissues [30]. However, because HC loss occurs in a slow and progressive fashion in this model it is not suitable for the study of early developmental angiogenesis. Several transcription factor knock out mice, e.g., Ptf1, Prox1, and Foxn4, display HC specification defects [31]. Of these mouse lines, *Foxn4* ^-/-^ mice are the only model that is not lethal at birth and *Foxn4* ^*-/-*^ retinas display a complete absence of HCs and a reduced number of ACs [21]. However, whether these changes affect retinal vascular development is not known. Although the postnatal viability of *Foxn4* ^-/-^ mice is impaired, we were able to analyze mice from P0-21 for vascular defects. The P10 WT OPL was vascularized as expected, however intraretinal vessels were completely absent in *Foxn4* ^-/-^ littermates at this stage (Fig 4A–4C). By P14, WT mice displayed two well-developed intraretinal vascular plexuses in the IPL and OPL, while only a single, sparse, ectopic plexus had formed in *Foxn4* ^-/-^ littermates (Fig 4D–4F). At P21, vessels in the ectopic intraretinal plexus (Fig 4G–4I) were localized within or on either side of nuclei-free gaps (Fig 4H, right panel) that had formed in the INL of *Foxn4* ^-/-^ retinas. To confirm that the location of the intraretinal plexus was ectopic, we localized vessels relative to SOX9 positive Müller glia nuclei, PKCalpha positive rod bipolar cells, and Recoverin positive photoreceptors. Capillaries were found in a range of locations within the inner portion of the fused ONL/INL, but were generally displaced towards the inner boundary of the retina. Capillaries frequently overlapped with Müller glia nuclei (Fig 4G), which are positioned below rod bipolar cell bodies, and staining for photoreceptor nuclei further confirmed a shift in capillary location towards the inner boundary of the retina both at P14 and P21 (Fig 4I).

Analysis of retinal whole mounts showed that vascular defects in *Foxn4* ^-/-^ retinas predominantly affected the intraretinal capillaries. Vascular development in the NFL was delayed at P10 (Fig 4J–4L) but by P14, the NFL vasculature appeared less affected than the intraretinal capillaries. The ectopic vascular plexus in *Foxn4* ^-/-^ retina was incomplete and poorly developed at P14 (Fig 4M–4O).

The finding that intraretinal capillaries are poorly developed and ectopic in *Foxn*4^-/-^ retinas demonstrates that Foxn4-dependent neuronal differentiation is required for normal retinal vascular morphogenesis. These results highlight that interneurons have key roles in instructing intraretinal capillary formation.

### Altered vascular lesion formation in *Foxn4* ^-/-^;*Vldlr* ^*-/-*^ mice

**Fig 5:**
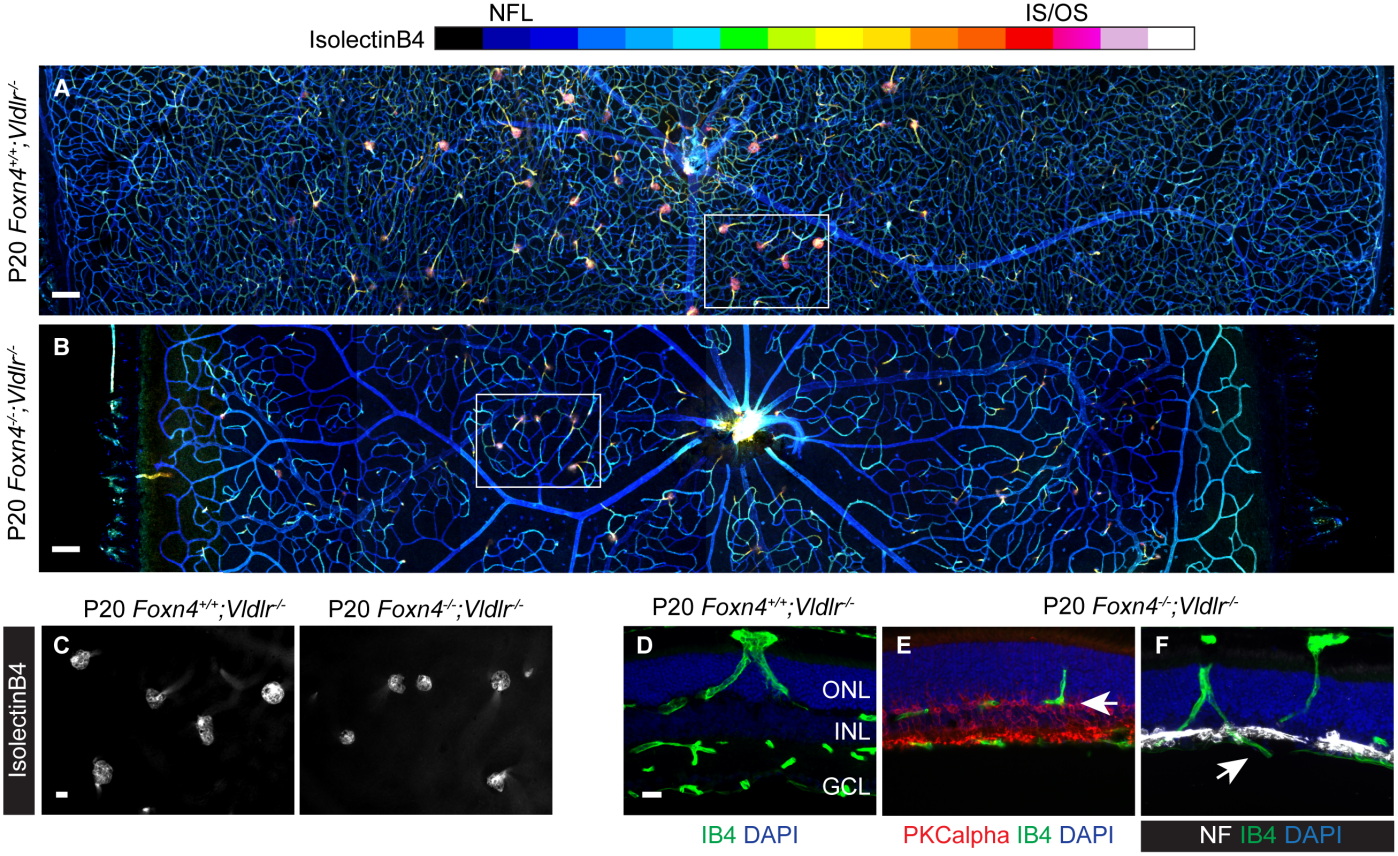
Pathological angiogenesis in *Foxn4* ^-/-^ *;Vldlr* ^-/-^ mice. (**A-B**) Retinal whole mounts stitched from confocal depth projections (color coded). In *Vldlr* ^-/-^ mice, vascular lesions occur predominantly in the central retina. In the *Foxn4* ^-/-^ *;Vldlr* ^-/-^ DKO retina, lesions are seen in both the central and peripheral retina. (**C**) Epifluorescent images taken from the outer retinal side of the boxed areas in A and B show vascular lesion heads. (**D-F**) SRV originates from OPL capillaries in the *Vldlr* ^-/-^ retina, while vascular lesions stem from either the single intraretinal plexus (arrow in E, relative to bipolar cell marker PKCalpha) or from the superficial plexus (arrow in F, relative to the HC and ganglion cell marker neurofilament) in the *Foxn4* ^-/-^ *;Vldlr* ^-/-^ retina. Neurofilament staining confirms the absence of HCs. GCL, ganglion cell layer; ONL, outer nuclear layer; OPL, outer plexiform layer. Scale bars: A-B, 100 μm; C-F, 20 μm.

In order to determine the role of mistargeted HC neurites in SRV we generated *Foxn4* ^-/-^;*Vldlr* ^*-/-*^ mice. Analysis of P20 retinal whole mounts showed that *Foxn4* ^-/-^;*Vldlr* ^*-/-*^ mice, like *Foxn4* ^-/-^ mice, displayed a single, poorly developed intraretinal vascular plexus. Importantly, *Foxn4* ^-/-^;*Vldlr* ^*-/-*^ mice developed a significant number of vascular lesions directed towards the subretinal space (Fig 5B and 5C). SRV in *Vldlr* ^*-/-*^ mice developed predominantly in the central retina and originated from OPL capillaries, however, in *Foxn4* ^-/-^;*Vldlr* ^*-/-*^ mice lesion formation was more homogenous across the retina (Fig 5A and 5B) and vascular lesions originated from either the superficial vascular plexus in the NFL (Fig 5E) or from the ectopic intraretinal plexus (Fig 5F).

Our analysis of *Foxn4* ^-/-^;*Vldlr* ^*-/-*^ mice shows that vascular lesions can form in the absence of HCs and mistargeted HC neurites. Conversely, we observed HC neurite mistargeting in *Vldlr* ^*-/-*^ mice at P6.5 before the deep layers of the retina are vascularized (Fig 3A). Thus, HC neurite mistargeting and SRV appear to be independent phenotypes despite the high degree of association between vascular lesions and mistargeted neurites in *Vldlr* ^*-/-*^ mice (Fig 3).

### The OPL and IPL are vascularized in an inverse order in *Vldlr* ^*-/-*^ mice

**Fig 6:**
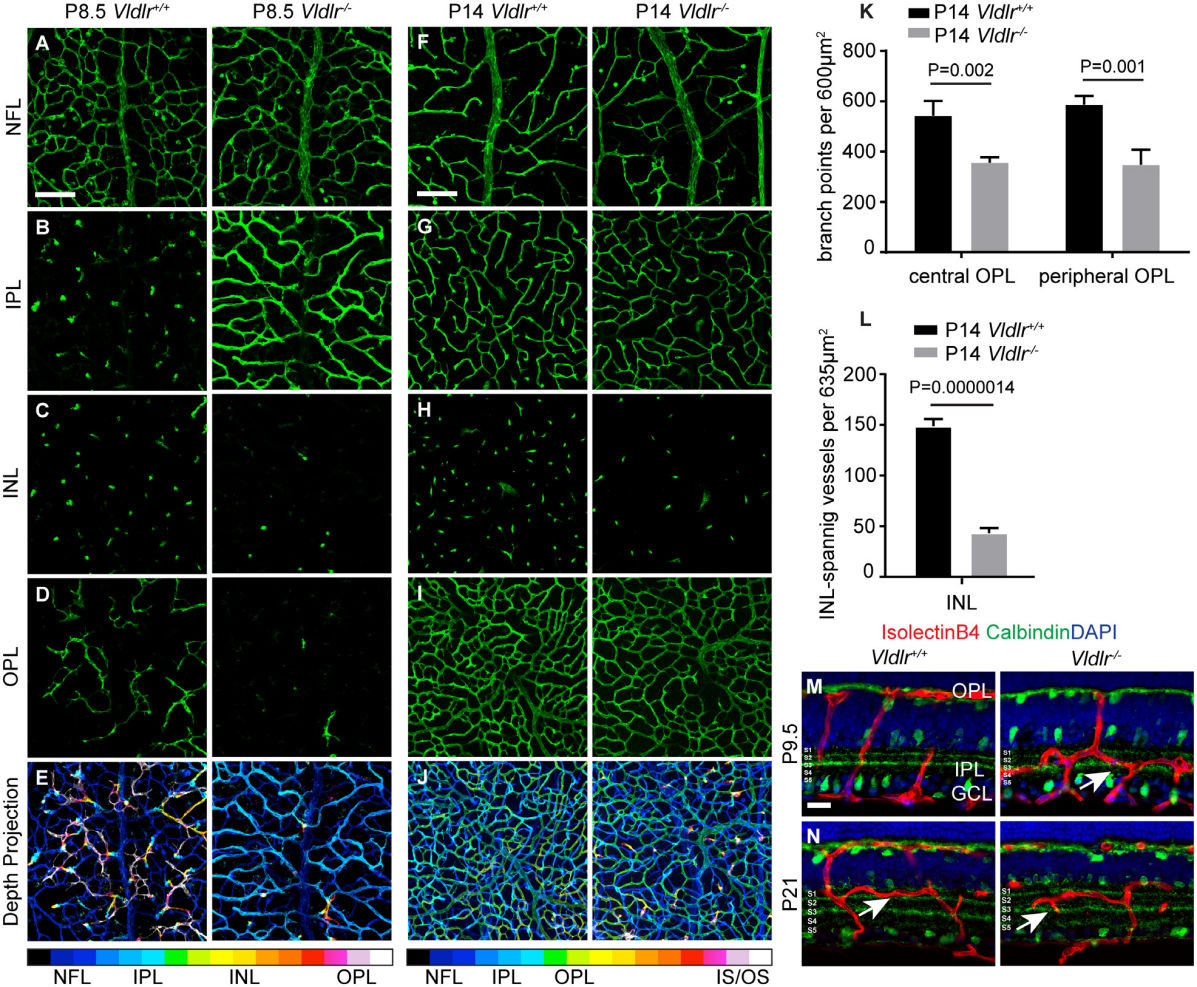
Inverse order of intraretinal capillary plexus formation in *Vldlr* ^-/-^ mice. (**A-D**) Confocal z-projections of isolectinB4 stained retinal vasculature in the indicated retinal layers. In WT mice the OPL is vascularized first, at P8.5 vertically oriented vessels pass through the IPL and INL to form a plexus in the OPL. In *Vldlr* ^-/-^ mice, the IPL is vascularized before the OPL. (**E**) Color-coded confocal depth projection. (**F-J**) By P14, *Vldlr* ^-/-^ mice have formed a plexus in the OPL, but few vertically oriented vessels connect the IPL and OPL network. The OPL plexus is less dense. (**K**) Quantification of branch points in the OPL plexus reveals a significant reduction in branch point number both in the central and peripheral retina in *Vldlr* ^-/-^ mice (n = 3–4, mean +STDEV shown). (**L**) Quantification of INL spanning vertical blood vessels reveals a highly significant reduction in P14 *Vldlr* ^-/-^ retinas. (**M, N**) Inverse order of intraretinal plexus development revealed on retinal sections. In addition to the altered timing of intraretinal plexus development, capillaries in *Vldlr* ^-/-^ retinas are also subtly mislocalized. In P21 WT retinas, the IPL capillaries are directly juxtaposed to cell bodies in the INL, whereas capillary location is shifted towards the inner portions of the IPL in *Vldlr* ^-/-^ retinas (white arrows). The capillary location was evaluated relative to calbindin positive stratified neurites. This mislocalization was confirmed in virtually all *Vldlr* ^-/-^ retinal sections used in this study, see also Fig 7. GCL, ganglion cell layer; INL, inner nuclear layer, IPL, inner plexiform layer, IS/OS, inner and outer photoreceptor segments; NFL, nerve fiber layer; OPL, outer plexiform layer. Scale bars: A-J, 100 μm; M-N, 20 μm.

To extend our phenotypic analysis of the VLDLR model of SRV we investigated vascular development at early postnatal stages. In normal murine retinal vascular development, vertical vascular sprouts emerge from the NFL vasculature and populate the OPL between P8 and P12, then the IPL becomes vascularized [3]. While the exact timing of intraretinal capillary development can be influenced by genetic background and other factors, the OPL is always vascularized before the IPL. When we analyzed vascular development in the NFL, IPL, and OPL using confocal microscopy, we found that the sequence of vascular development was strikingly altered in *Vldlr* ^*-/-*^ mice. In P8.5 WT retinas, as expected, the IPL was devoid of a capillary plexus, and only vertically oriented sprouts passed through the IPL and INL in order to form the first intraretinal plexus in the OPL. In stark contrast, we found that *Vldlr* ^*-/-*^ littermates displayed the opposite order of intraretinal plexus formation. At P8.5, a dense vascular plexus had formed in the IPL, while only a few vertically oriented sprouts had passed to an OPL still largely devoid of capillaries (Fig 6B–6E). To our knowledge, no mouse mutant that phenocopies this inverse order of intraretinal plexus formation has been reported. By P14, the OPL vasculature had formed in both WT and *Vldlr* ^*-/-*^ mutant retinas (Fig 6G–6J), however, branch point analysis (a metric used to quantitatively assess vascular density) revealed that the OPL vasculature remained sparser in the mutant retina (34% and 41% reduction in vascular density in the central and peripheral retina, respectively, Fig 6K). In addition, significantly fewer vertical sprouts passed through the INL in the central mutant retina as compared to WT littermates (71% reduction, Fig 6L). While intraretinal vascular development in *Vldlr* mutant mice was strongly altered, we found that the NFL vasculature in the central retina appeared largely normal (Fig 6A and 6F).

Analysis of the vascular networks in the IPL and OPL using P9.5 retinal sections further confirmed that the IPL capillary bed forms before the OPL is vascularized in *Vldlr* ^*-/-*^ retinas (Fig 6M). In addition, relative to Calbindin—a marker for ACs in the INL and displaced ACs in the GCL—the IPL capillaries in mutant retinas were mislocalized. IPL capillaries in P21 WT retinas were tightly associated with cell bodies at the IPL/INL border in stratum S1 (Fig 6N left panel), whereas capillaries in the mutant retina at P9.5 and at P21 were consistently detected in a position shifted towards the inner portion of the IPL and were often found in strata S2–S3 (Fig 6M and 6N, right panels). This subtle mislocalization of IPL capillaries in *Vldlr* ^*-/-*^ mice may represent ectopic vascularization, or alternatively, a significant expansion of the intersublaminar plexus [32].

**Fig 7:**
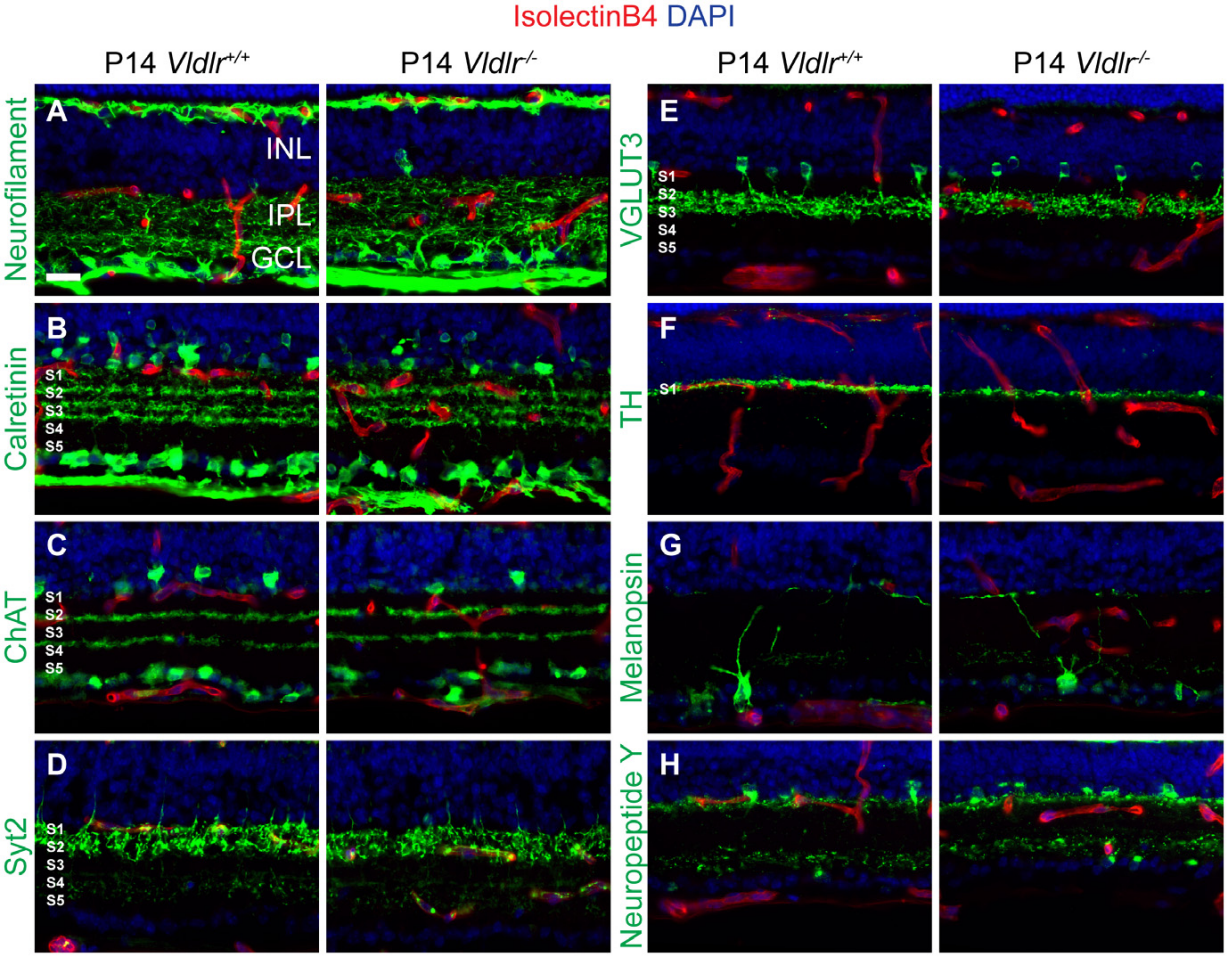
The IPL vascular plexus is incorrectly positioned, but IPL neurite stratification appears normal in *Vldlr* ^-/-^ retinas. (**A-H**) While most IPL capillaries are juxtaposed to the cell bodies of the INL in WT retina, capillaries in *Vldlr* ^-/-^ retinas are frequently and consistently mislocalized towards the inner surface of the retina. Analysis of neurite stratification of several classes of amacrine cells and one class of ganglion cells shows no defect in neurite stratification. GCL, ganglion cell layer; INL, inner nuclear layer; IPL, inner plexiform layer; S 1–5, strata 1–5. Scale bar: 20 μm.

Because vascular defects in the outer retina are associated with neurite mistargeting in *Vldlr* ^*-/-*^ mice, we further analyzed IPL stratification and the location of IPL capillaries. Co-staining of isolectinB4 (to visualize blood vessels) with neurofilament (ganglion cell processes), calretinin (ACs and ganglion cells), choline acetyl transferase (ChAT, cholinergic ACs), Synaptotagmin 2 (Syt2, cone OFF bipolar cells), vesicular glutamate transporter type 3 (VGLUT3, glutamatergic ACs), tyrosine hydroxylase (TH, dopaminergic ACs), Melanopsin (intrinsically photosensitive retinal ganglion cells), or Neuropetide Y (NPY positive ACs) confirmed that the location of IPL capillaries is altered in *Vldlr* ^*-/-*^ mice (Fig 7A–7H). None of the markers of neurite stratification in the IPL showed detectable structural differences between WT and mutant retinas. Our finding that the two intraretinal capillary plexuses form in an inverse order and that the IPL capillary position is shifted in *Vldlr* ^*-/-*^ mice shows that SRV occurs against the backdrop of severe defects in vascular development in the *Vldlr* ^*-/-*^ model.

### Retinal vascular proliferation in *Vldlr* ^*-/-*^ mice is increased in the second but not the first postnatal week

**Fig 8:**
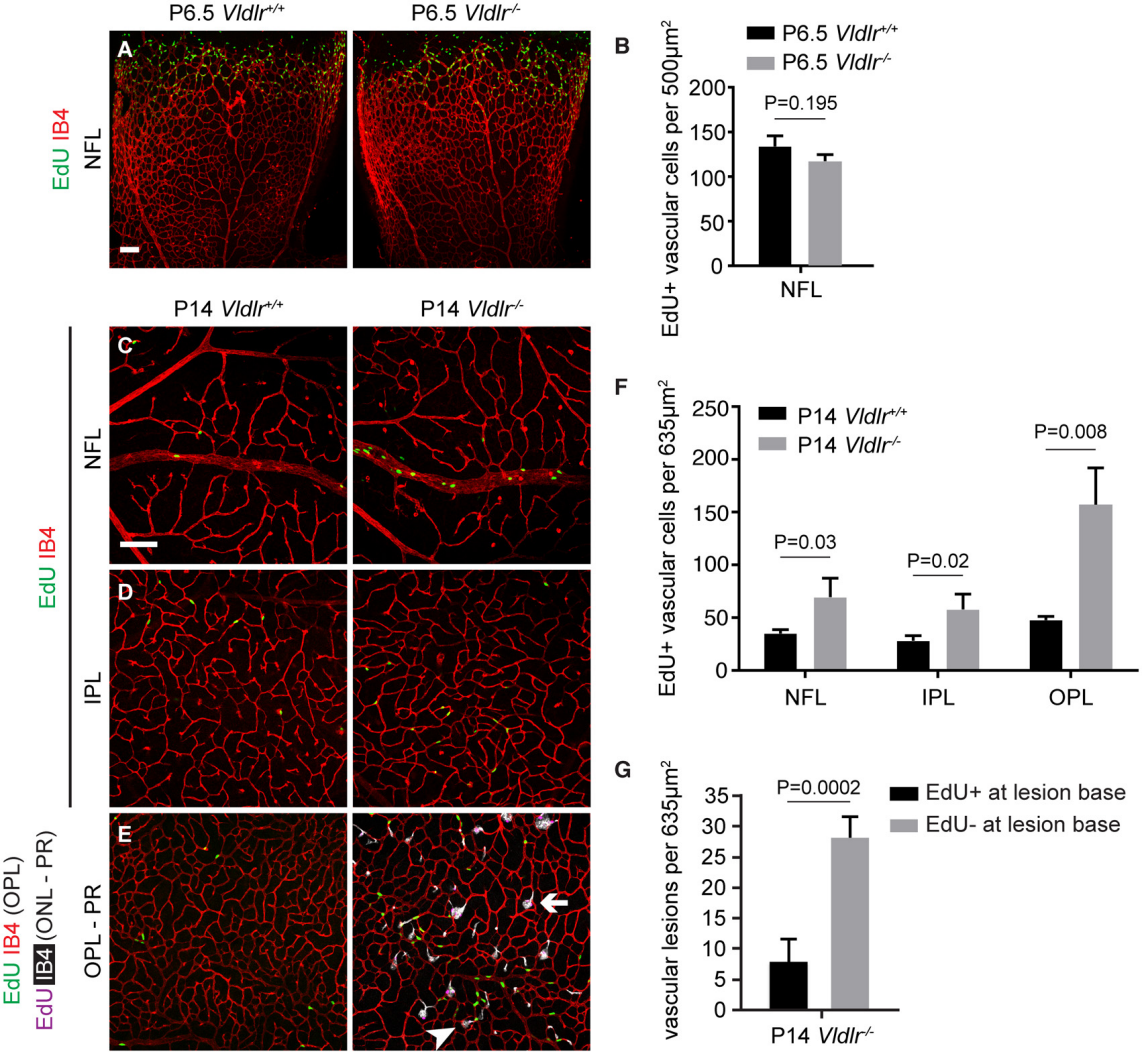
Onset of increased vascular cell proliferation in *Vldlr* ^-/-^ retinas is in the second postnatal week. (**A, C-E**) Confocal images of the indicated vascular plexuses of mice injected with EdU for quantification of proliferating cells. (**E**) shows proliferating vascular cells at two depth levels: EdU positive cells and vasculature in the OPL are displayed in green and red, respectively. EdU positive cells and vascular lesions are displayed in magenta and white, respectively. The image shows lesions without EdU positive cells at the lesion base (arrow) as well as lesions with EdU positive cells at the lesion base (arrowhead). **(B**) Vascular development and cell proliferation appear unaltered at P6.5. (**C-F**) Proliferation of vascular cells is increased at P14, especially in the OPL (n = 3, mean +STDEV shown). (**G**) Quantification of the number of vascular lesions with or without EdU positive cells at the lesion base in the OPL (n = 4, mean +STDEV shown). NFL, nerve fiber layer; INL, inner nuclear layer; OPL, outer plexiform layer; ONL, outer nuclear layer; PR, photoreceptor layer. Scale bar: 100 μm.

Abnormalities in early *Vldlr* ^*-/-*^ vascular development may play important roles in subsequent SRV in these mice. To determine if developmental phenotypes are associated with changes in vascular cell proliferation, we used the thymidine analog 5-ethynyl-2’-deoxyuridine (EdU) to measure active DNA synthesis in vascular cells. At P6.5, vascular cell proliferation at the vascular front in WT and mutant retinas was virtually equal, and the superficial vascular plexus in *Vldlr* ^*-/-*^ mice was morphologically indistinguishable from WT littermates (Fig 8A and 8B). However, at P14, proliferation in all three vascular layers was significantly increased. This difference was especially noticeable in the OPL, where an approximately 3-fold increase in proliferation relative to WT littermates was observed (Fig 8C–8F). Proliferating vascular cells did not specifically cluster around the origins of vascular lesions in the OPL (Fig 8G), indicating that lesion formation is not due to foci of increased vascular proliferation. Proliferating cells were, however, frequently detected in lesion heads, i.e., at the level of the subretinal space at P14 (S1 Video).

Our analysis demonstrates that the inverse order of intraretinal capillary bed formation has a critical role in triggering subsequent vascular defects, including delayed vascularization of the OPL. The delayed OPL vascularization in *Vldlr* ^*-/-*^ mice (Fig 6) is followed by a delayed onset of endothelial quiescence (Fig 8). Thus, developmental vascular defects and increased vascular cell proliferation may contribute to the development of SRV in *Vldlr* ^*-/-*^ mice.

## Discussion

Our phenotypic analysis places SRV in *Vldlr* ^-/-^ mice into a complex developmental context. We observed both neuronal and vascular phenotypes before the onset of SRV, but we detected strong expression of *Vldlr* mRNA only in the neural retina (Fig 2), suggesting that VLDLR functions in neural cells (though perhaps not exclusively). We found that HC neurite mistargeting precedes vascular lesion formation in the *Vldlr* ^*-/-*^ model of SRV, and that SRV is associated with mistargeted neurites (Fig 3). However, despite the association between vascular lesions and mistargeted HC neurites, we found that HCs are not required for SRV in *Foxn4* ^-/-^;*Vldlr* ^*-/-*^ mice (Fig 5). Conversely, it is unlikely that neurite phenotypes are caused by vascular defects because aberrant neurite stratification in *Vldlr* ^*-/-*^ retinas can be detected as early as P6.5, before the retinal vasculature appears atypical or is in contact with HCs. More likely, HC neurite mistargeting and SRV occur as separate consequences of a pathologically changed neuroretina.

The neurovascular interaction that we describe here between intraretinal blood vessels and HCs is novel. During development, neurovascular congruence has been described in the peripheral nervous system, e.g., between peripheral nerves and the vasculature [33, 34], however, interactions between subcellular processes of central neurons and pathological retinal vasculature have not been studied. In light of the novel neurovascular interaction between HC neurites and SRV it is important to consider how frequently SRV occurs in the context of neurite mistargeting in other mouse models of SRV and in human disease. It is interesting to note that in the Ccl2/Cx3cr1/rd8 model, which develops vascular lesions originating from both the choroidal vasculature [35] and the retinal vasculature [36], HC and bipolar cell neurite mistargeting into the ONL has been reported [37]. It is unclear, however, if neurites are closely associated with SRV in these mice. It is also not known if mistargeted neurites contribute to SRV in human disease, e.g., in RAP, proliferative macular telangiectasia, or choroidal neovascularization with retinal anastomosis. Reduced neurotransmission at the photoreceptor triad synapse triggers HC and bipolar cell neurite sprouting [38–44] and previous studies have suggested that declining photoreceptor function causes neurite mistargeting in elderly human donors and in aged rodents [45–48]. Therefore, SRV may commonly develop in a context where retinal neurons extend aberrant neurites into the ONL and it will be important to determine if the mistargeted neurites have roles in facilitating, stabilizing, or localizing SRV.

The neurite mistargeting phenotype in *Vldlr* ^*-/-*^ mice appears to be distinct from similar phenotypes in other mouse models. Mutations that alter photoreceptor neurotransmission also cause bipolar cell neurite mistargeting and ectopic glutamatergic synaptogenesis in the ONL [38–44]—neither of which we observed in *Vldlr* ^*-/-*^ mice (Fig 3N and 3O). Mistargeted HC neurites in *Sema6A* and *PlexinA4* mice [49] have a different microscopic appearance than those observed in *Vldlr* ^*-/-*^ mice, and these mice also display dopaminergic AC and melanopsin positive ganglion cell stratification defects in the IPL, which we did not observe in *Vldlr* ^*-/-*^ mice (Fig 7).

A central conclusion of the present study is that HCs and ACs play important roles in normal retinal angiogenesis. Although interactions between astrocytes, retinal ganglion cells, and blood vessels have been previously studied [18], it is incompletely understood how the neuroretina instructs retinal vascular morphogenesis. Our finding that loss of Foxn4-dependent HC and AC differentiation causes severe vascular phenotypes provides important evidence that additional neuronal populations play a major role in normal angiogenesis (Fig 4). Our observations raise questions about which pro-angiogenic factors, cell-cell adhesion molecules, and matricellular proteins are expressed by HCs and ACs and how the expression of these factors is controlled. The associations of mistargeted HC neurites and SRV suggest that HCs may normally provide guidance cues that help localize capillaries in the laminated retina.

Our developmental analysis also revealed novel retinal vascular phenotypes in *Vldlr* ^-/-^ mice that precede SRV. The intermediate capillary plexus develops in the IPL before the formation of the outer plexus in the OPL, and intraretinal capillary morphogenesis is severely altered before the onset of vascular lesion formation (Figs 6 and 7). Importantly, these early phenotypes are followed by increased vascular proliferation, particularly in the OPL. Interestingly, Inhibition of VEGF signaling, Norrin/Frizzled4 signaling, and Ras activity partially or fully suppress SRV in *Vldlr* ^*-/-*^ mice [10, 11, 50, 51], possibly by normalizing the increased vascular proliferation we observed *in vivo*.

Our analysis also showed that OLM disruption is an important intermediate step in the progression from early phenotypes to late phenotypes. We found that blood vessels in the subretinal space were leaky despite the fact the SRV originated from non-fenestrated OPL capillaries (i.e., endothelial cells that lack substantial PLVAP expression). This finding is consistent with a previous report that documents increased PLVAP expression in *Vldlr* ^*-/-*^ mice [10], and suggests that SRV leakiness is not determined by the origin of SRV vasculature (choroidal vs. retinal vasculature), but appears to be a property of blood vessels in the subretinal space. This could be due to the absence of ligands activating the Norrin/Frizzled4 pathway, a key driver of blood-retina-barrier formation and PLVAP repression in endothelial cells [52–54], in this location. Our analysis also highlights similarities and differences between SRV in the *Vldlr* ^*-/-*^ animal model and in human instances of SRV. While the consequences of OLM disruption are likely similar in the *Vldlr* ^*-/-*^ model and in human SRV with retinal origin, our finding that SRV in the *Vldlr* ^*-/-*^ model develops in the context of complex developmental phenotypes indicates that there are important differences.

Given that mutations in photoreceptor-specific genes can cause HC neurite mistargeting and that photoreceptors express anti-angiogenic molecules [55], it will be important to determine if VLDLR is functionally required in photoreceptors. Instead of a direct role in cell-cell signaling, VLDLR loss of function phenotypes could result primarily from its role in lipoprotein uptake and metabolism. For example, a metabolic imbalance in photoreceptors could cause changes in expression or function of factors that control both neurite stratification and vascular morphogenesis. One factor that could affect both the retinal vasculature and HC neurites are ROS. ROS accumulate in *Vldlr* ^*-/-*^ retinas [11] and pharmacological suppression of ROS reduces SRV [12–14, 56], but whether ROS are involved in HC neurite mistargeting or the inverse order of intraretinal plexus formation is not known. Alternatively, defects in photoreceptors and/or other retinal neurons may alter the function of a guidance cue that repels both endothelial cells and HC neurites [57].

## Supporting Information

S1 ARRIVE Checklist (PDF)

S1 Fig Schematic horizontal section through the murine retina. (**A**) The retina is laminated and contains three nuclear layers, the ganglion cell layer (GCL), inner nuclear layer (INL), and outer nuclear layer (ONL). Synapses form in the inner plexiform layer (IPL) and outer plexiform layer (OPL). Other structures: outer limiting membrane (OLM) and retinal pigment epithelium (RPE) with Bruch's membrane. Simplified circuit: photoreceptors (purple) synapse to bipolar cells (green), which control the activity of ganglion cells, the retinal output neurons (yellow). Horizontal cells (black) and amacrine cells (red) modulate the circuit. Not shown: Neurite strata (layers) in the IPL formed by specific connectivity of different classes of bipolar cells, amacrine cells, and ganglion cells. (**B**) Three layers of retinal vasculature. The outer retina is also supplied by choroid capillaries. (**C**) Vascular lesions in the *Vldlr* mutant mouse.
(TIF)

S2 Fig Branched DNA ISH supporting information. **(A)** Validation of the *Vldlr* exon5 probe set (probe^Vldlr_exon5^) in HeLa cells transfected with the indicated constructs. (**B**) Transfected constructs express at similar levels as shown by immunostaining for the HA tagged protein. (**C-G**) Branched DNA ISH on ocular tissues as indicated.
(TIF)

S3 Fig No horizontal cell neurite mistargeting in *Reelin* ^-/-^ mice. (**A-B**) Staining of rod bipolar cells with PKCalpha confirms occasional ectopic synaptic terminals in *Reelin* ^-/-^ mice. (**C**) No neurofilament positive mistargeted HC neurites were seen in adult *Reelin* ^-/-^ mice.
(TIF)

S1 Video P14 vascular lesion with EdU positive cells. Confocal 3-D rendering spanning an area of the outer retina from the OPL to the photoreceptor layer in a *Vldlr* ^*-/-*^ retina. IB4 stained blood vessels (green) and a single vascular lesion with two EdU positive cells (red) in the lesion head are shown.
(AVI)
