# Supplementary figures and images for: Neurite Mistargeting and Inverse Order of Intraretinal Vascular Plexus Formation Precede Subretinal Vascularization in Vldlr Mutant Mice
Source: PLoS One. 2015 Jul 15;10(7):e0132013. doi: 10.1371/journal.pone.0132013 (PMC4503745; doi:10.1371/journal.pone.0132013)

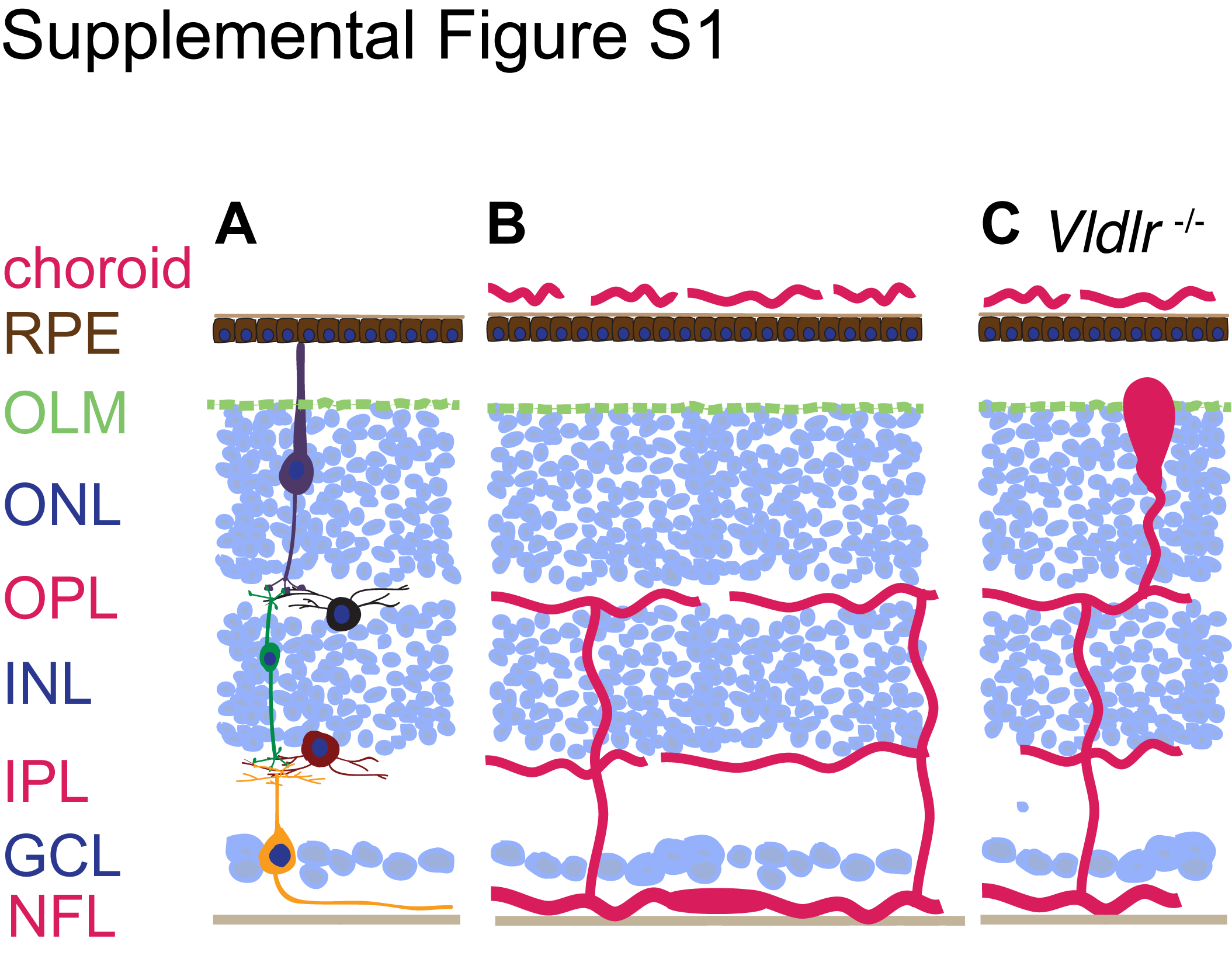

Supplement: S1 Fig — (A) The retina is laminated and contains three nuclear layers, the ganglion cell layer (GCL), inner nuclear layer (INL), and outer nuclear layer (ONL). Synapses form in the inner plexiform layer (IPL) and outer plexiform layer (OPL). Other structures: outer limiting membrane (OLM) and retinal pigment epithelium (RPE) with Bruch's membrane. Simplified circuit: photoreceptors (purple) synapse to bipolar cells (green), which control the activity of ganglion cells, the retinal output neurons (yellow). Horizontal cells (black) and amacrine cells (red) modulate the circuit. Not shown: Neurite strata (layers) in the IPL formed by specific connectivity of different classes of bipolar cells, amacrine cells, and ganglion cells. (B) Three layers of retinal vasculature. The outer retina is also supplied by choroid capillaries. (C) Vascular lesions in the Vldlr mutant mouse. (TIF) [file pone.0132013.s002.tif]

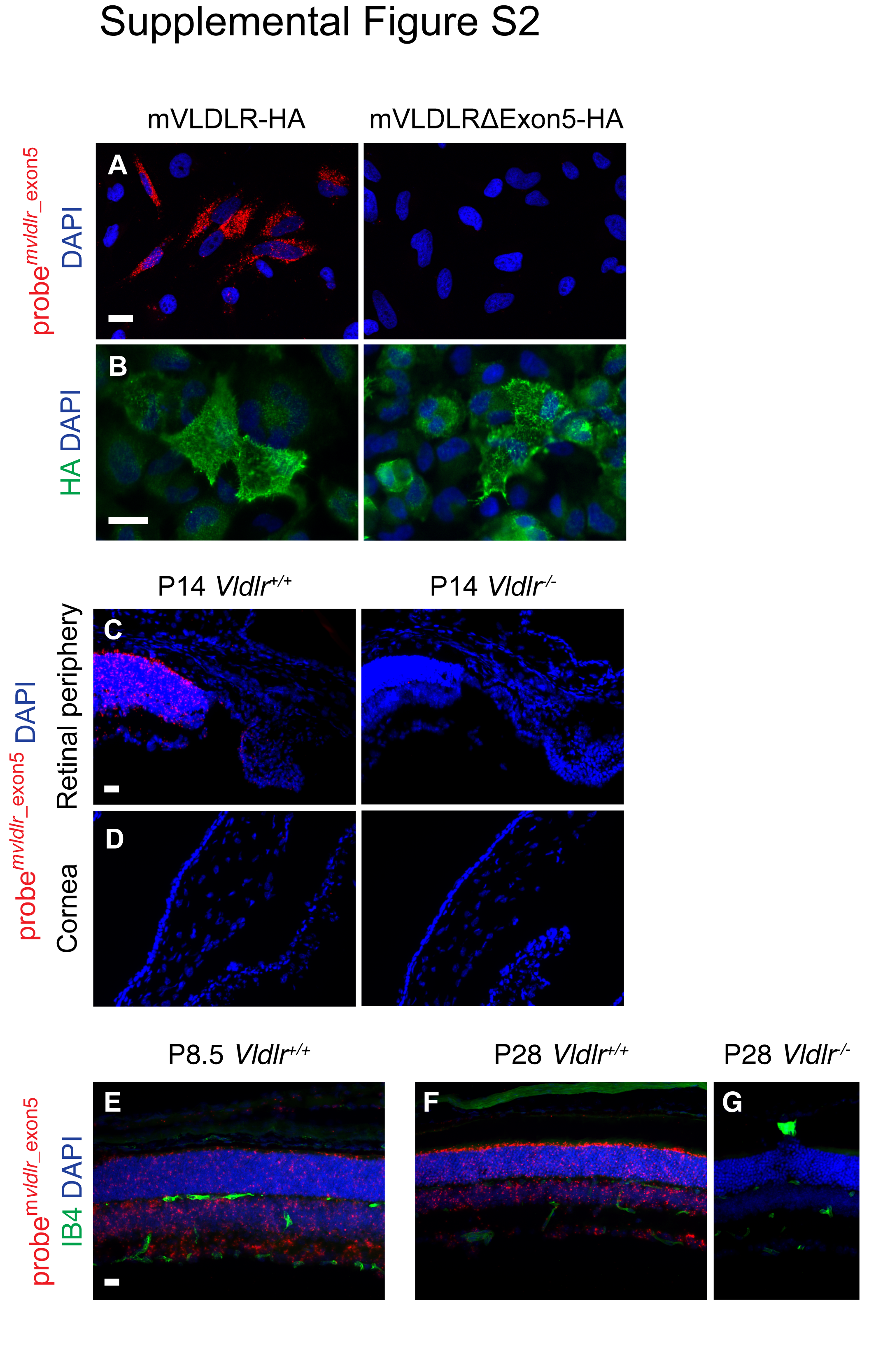

Supplement: S2 Fig — (A) Validation of the Vldlr exon5 probe set (probeVldlr_exon5) in HeLa cells transfected with the indicated constructs. (B) Transfected constructs express at similar levels as shown by immunostaining for the HA tagged protein. (C-G) Branched DNA ISH on ocular tissues as indicated. (TIF) [file pone.0132013.s003.tif]

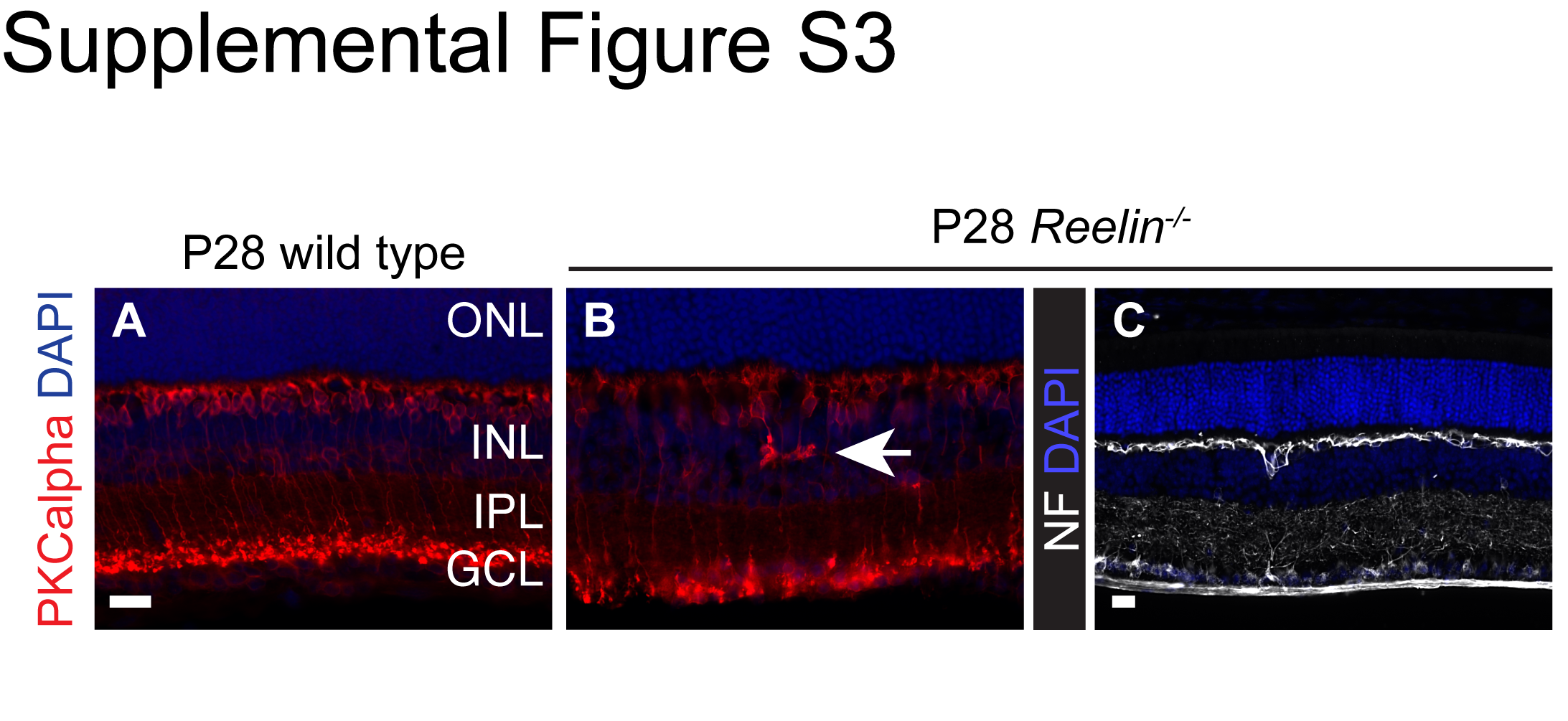

Supplement: S3 Fig — (A-B) Staining of rod bipolar cells with PKCalpha confirms occasional ectopic synaptic terminals in Reelin -/- mice. (C) No neurofilament positive mistargeted HC neurites were seen in adult Reelin -/- mice. (TIF) [file pone.0132013.s004.tif]
